# Filter survival time and requirement of blood products in patients with severe sepsis receiving drotrecogin alfa (activated) and requiring renal replacement therapy

**DOI:** 10.1186/cc7163

**Published:** 2008-12-18

**Authors:** Luigi Camporota, Eleonora Corno, Eleonora Menaldo, John Smith, Katie Lei, Richard Beale, Duncan Wyncoll

**Affiliations:** 1 Adult Intensive Care Unit, Guy's and St Thomas' NHS Foundation Trust, St Thomas' Hospital, 1st Floor East Wing, Lambeth Palace Road. London SE1 7EH, UK

## Abstract

**Introduction:**

Drotrecogin alfa (activated) (DrotAA) is licensed in the United States and the European Union for the treatment of severe sepsis with multiple organ failure. Patients with severe sepsis on renal replacement therapy (RRT), who typically receive additional anticoagulation to prevent circuit clotting, may be at higher risk of bleeding when DrotAA is administered in addition to standard anticoagulation. However, the effects of DrotAA on filter duration in the absence of additional anticoagulation have not been established. The aim of this study was to analyse the filter survival time (FST), and to quantify the requirement of packed red cells (PRC) and blood products during DrotAA infusion.

**Methods:**

This was a single-centre, retrospective observational study conducted in an adult intensive care unit (ICU). Thirty-five patients with severe sepsis who had received both RRT and DrotAA were identified, and all relevant clinical and laboratory data were retrieved from the departmental electronic patient record. We compared haemofilter parameters, requirement of blood products and haemodynamic data recorded during RRT and the infusion of DrotAA with those recorded on RRT with standard anticoagulation after the DrotAA infusion had been completed (post-DrotAA).

**Results:**

The proportion of filter changes due to filter clotting was similar during DrotAA infusion and with conventional anticoagulation post-DrotAA infusion. There was no difference in the FST and filter parameters during DrotAA in the presence or absence of additional anticoagulation with heparin or epoprostenol. A similar proportion of patients required red cell transfusion, although a greater proportion of patients received platelet and fresh frozen plasma during DrotAA infusion compared with the post-DrotAA period with no difference between medical and surgical patients.

**Conclusions:**

Additional anticoagulation during DrotAA infusion does not appear to improve FST. The use of DrotAA in patients with severe sepsis requiring RRT is safe and is not associated with an increased need for PRC transfusion or major bleeding events.

## Introduction

Drotrecogin alfa (activated) (DrotAA; Xigris, Eli Lilly & Co., Indianapolis, USA), a recombinant human activated protein C, can reduce mortality in patients with severe sepsis [1]. Data from long-term follow-up studies [2] and from international and national registries have also confirmed that the effects of DrotAA on survival seen in clinical practice are consistent with those seen in randomised trials [1,3-8]. However, despite the beneficial outcome data and acceptable safety profile, intensivists are often reluctant to prescribe DrotAA in certain groups of patients (e.g., patients who have undergone surgery, patients with coagulopathy or those who receive anticoagulants) because the anticoagulant properties of DrotAA might increase the risk of bleeding, particularly in the presence of clotting abnormalities associated with sepsis.

Anticoagulants are generally given to patients requiring renal replacement therapy (RRT) to prolong the duration of the filter and to prevent damage and loss of platelets and erythrocytes in the clotted circuit. However, the risk of bleeding and the lifespan of the RRT circuit in patients who continue anticoagulation while receiving DrotAA compared with patients who receive DrotAA alone is not known. Therefore, the two main issues concerning the use of DrotAA during RRT are: the safety of DrotAA in renal failure requiring RRT; and the need for additional anticoagulation to preserve filter function and prolong filter survival.

Pharmacokinetic data demonstrate that DrotAA is not eliminated by haemofiltration or dialysis, and its serum concentration and drug half-life are similar in patients with or without renal failure; therefore, no dose adjustment is required [9]. However, no specific data are available on the safety of DrotAA, duration of the RRT circuit survival, bleeding events leading to red cell transfusion or blood products for the correction of clotting abnormalities during RRT in septic patients who receive DrotAA.

The aims of our study were: to analyse the filter survival time (FST) during DrotAA infusion – in the presence or absence of additional anticoagulation compared with the 96 hours post-DrotAA infusion – with standard anticoagulation, while still on RRT; and to quantify the use of packed red cell (PRC) and blood products in patients with acute kidney injury requiring RRT treated with DrotAA.

## Materials and methods

### Patients

This was a retrospective, single-centre study of patients with severe sepsis admitted to the adult intensive care unit (ICU) at St Thomas' Hospital in London, UK. We included all patients who received DrotAA for severe sepsis with two or more organs in failure and with acute kidney injury requiring RRT between 2002 and 2006. The selection of patients eligible for DrotAA was made in accordance to local protocols and guidelines [see Additional data file 1]. In order to achieve the aims of the study, we specifically selected patients who received a full course of DrotAA and a minimum of eight days of RRT.

Patients' demographic information, markers of disease severity, biochemical and haematological data, details of anticoagulation and RRT – including filter pressure parameters – and quantity and timing of blood products transfused were obtained by searching the departmental electronic patient record (CareVue Classic, Philips Medical Systems, Böblingen, Germany). The modality of RRT used was continuous venous-venous haemofiltration.

The aim of the study was to compare the proportion of filter changes secondary to circuit clotting, the FST and the red cells and blood product transfusion in the same patients during the infusion of DrotAA and post-DrotAA, provided they remained on RRT.

A further comparison of the outcome parameters was made during the DrotAA infusion period between patients who received DrotAA without any additional anticoagulation, and patients who received DrotAA with additional anticoagulation – heparin or epoprostenol. This study was considered by the National Research Ethics Service as 'service evaluation' and therefore did not require Research Ethics Committee review [10].

### Statistical analysis

Distribution of baseline variables was assessed using the Kolmogorov-Smirnov test. Differences in baseline variables between survivors and non-survivors were compared using a two-tailed t-test or Mann-Whitney U test for continuous data, and chi-squared or Fisher's exact test for qualitative data. The Kruskall-Wallis test was used for multiple comparisons.

Variables found to be associated with filter survival in an analysis of covariance were entered in a multivariate logistic backward-likelihood ratio regression analysis, to identify predictors of mortality at different end-points. The Hosmer and Lemenshow goodness-of-fit test was used to test the validity of the model. Analyses were performed using SigmaStat software v. 3.0 (Systat Software Inc, San Jose, CA, USA).

## Results

Patients' characteristics are shown in Table 1.

**Table 1:** Baseline characteristics of study population.

**Overall population**	
N	35
Age, years	65 ± 15
Male/Female	23/12
SOFA	13 ± 2.5
Organ dysfunction, n	4 ± 0.8
APACHE II	23 ± 5
ICU mortality, n (%)	14 (40)
Hospital mortality, n (%)	16 (45.7)
Patients with previous renal impairment*	11 (31%)
Medical, n (%)	15 (43%)
Surgical, n (%)	20 (57%)
Urea, mmol/L	13.5 ± 9.7
Creatinine, μmol/L	198.1 ± 108.4

Data are expressed as absolute number (n), percentage or mean ± SD.

* Impaired renal function was defined as patients receiving chronic dialysis or with documented elevated creatinine prior to current illness.

APACHE = Acute Physiology and Chronic Health Evaluation, ICU = intensive care unit, SOFA = Sequential Organ Failure Assessment.

### Effects of DrotAA on filter survival during renal replacement therapy in severe sepsis

During the entire study period, there were a total of 145 filter episodes, with a median (interquartile range (IQR)) = four (three to five) filter-episode per patient. During RRT DrotAA was temporarily interrupted in 8 of 35 (22.8%) patients in order to perform medical procedures (central venous catheter insertion/removal, n = 4; tracheostomy n = 1; removal of an intra-aortic balloon pump n = 3).

Although the median (IQR) number of filter episodes per patient was higher during the DrotAA infusion (89 during DrotAA; for 35 patients; average 2.5 filter episode per patient) compared with the post DrotAA period (56 post-DrotAA; for 24 patients; average 2.3 filter episode per patient) (median (IQR) = three (two to three) versus two (one to three), respectively; p = 0.01), the proportion of filters replaced because of a clotted circuit was similar during and after DrotAA infusion (35 of 89 (39.3%) during DrotAA versus 20 of 56 (35.7%) post-DrotAA) (Table 2). The majority of filter changes (60.7% during DrotAA and 63.6% post-DrotAA) occurred because of interruptions in RRT required to perform medical or surgical procedures, or radiological investigations – rather than filter clotting.

**Table 2:** Filter data

Total filter episodes (n)	145	
Total filters/patient median (range)	4 (1 to 8)	
Filters/patient on DrotAA median (range)	3 (1 to 4)	
Filters/patient post-DrotAA median (range)	2 (1 to 4)	
		
**Variable**	**DrotAA**	**Post-DrotAA**
Filter changes due to clotting	39.3%	36.4%
FST (hours)	22 (15 to 34)	16 (8 to 26)
Filters anticoagulated with heparin*	13	22
Filters anticoagulated with epoprostenol*	46	25
Filters anticoagulated with heparin and epoprostenol*	3	3
Filters with no additional anticoagulation*	27	6

Filter changes per patients and total filter episodes are expressed as absolute number. Filter survival time (FST) is expressed as median hours (interquartile range). * For all filter episodes, patients received drotrecogin alfa (activated) (DrotAA) in addition to the indicated anticoagulation during the DrotAA period. When no additional anticoagulation was administered filters were anticoagulated with DrotAA alone during DrotAA period.

The lifespan of the circuits changed because of a clotted filter was longer during DrotAA compared with the FST in the post-DrotAA (median (IQR) = 22 (15 to 34) hours versus 16 (8 to 26) hours); however, this difference was not statistically significant (p = 0.08), (Table 2). The median (IQR) filter life span for clotted versus non-clotted filters was 23.5 (11 to 37) hours and 22 (15 to 34) hours (p = 0.6) during DrotAA infusion and 16 (8 to 26) hours versus 13 (7 to 43) hours (p = 0.8) post-DrotAA, respectively. Comparisons of the other filter parameters during the 96-hour DrotAA infusion, and in the 96 hours post-DrotAA are shown in Table 3.

**Table 3:** Filter parameters.

**Variable**	**DrotAA median (IQR)**	**Post-DrotAA median (IQR)**	**p-value**
PFP_Max _(mmHg)	208 (142.7 to 247.7)	202 (147 to 258)	0.7
PFP_Min _(mmHg)	127 (91 to 169.5)	116 (76 to 161)	0.33
TMP_Max _(mmHg)	114 (88.7 to 151)	106 (79 to 154)	0.37
TMP_Min _(mmHg)	72 (45 to 101)	60 (48.5 to 73)	0.12
RP_Max _(mmHg)	155 (100.7 to 196)	140 (110 to 167)	0.21
RP_Min _(mmHg)	110(70.7 to 141)	94.5 (64 to 123.5)	0.05

Data are expressed as maximum and minimum median values (Interquartile range).

DrotAA = drotrecogin alfa (activated), PFP = pre-filter pressure, TMP = trans-membrane pressure, RP = return pressure.

The median (IQR) FST during DrotAA infusion was not significantly different between patients receiving DrotAA alone (27 filter episodes; 23 (15 to 35) hours), DrotAA and heparin (13 filter episodes; 21 (14 to 37) hours), DrotAA and epoprostenol (46 filter episodes; 23 (15 to 35) hours) or DrotAA with heparin and epoprostenol (3 filter episodes; 34 (11 to 36) hours) (p = 0.94, Kruskall-Wallis). There was also no difference in the FST between circuits anticoagulated with DrotAA alone versus all circuits anticoagulated with DrotAA plus other forms of anticoagulation (23.5 (11 to 35) hours) and (23 (11 to 35) hours; p = 0.7).

There was no difference in the values of the filter pressure parameters during DrotAA therapy and in the 96 hours post-DrotAA. The influence of clotting parameters on FST was also examined. There was no difference in the platelet count during DrotAA and post-DrotAA. There was, however, a significant difference in the maximum value of international normalized ratio (INR) (p = 0.047) and in the activated partial thromboplastin time (APTT) (p < 0.0001). Multivariate logistic regression analysis of the factors associated with filter clotting showed that the administration of other anticoagulants in addition to DrotAA and the levels of INR and APTT were not associated with filter clotting. The only predictive factor significantly, but weakly, associated with filter clotting during DrotAA, but not post-DrotAA, was the minimum value in platelet count: odds ratio = 1.007 (1.001 to 1.01); p = 0.002.

### Blood products requirement in patients treated with DrotAA

Differences in the haematological parameters during and after DrotAA are shown in Table 4. There was no difference in the proportion of patients requiring PRC transfusion during DrotAA infusion compared with the post-DrotAA period, on heparin (59% versus 50%; p = 0.8, Fisher's test). Although 3 of 35 (8.6%) patients required PRC transfusion of more than three units (median (IQR) 1375 (1357 to 1407) mL) during DrotAA infusion and none post-DrotAA, there was no statistical difference in the median (IQR) of PRC transfused (549 (292 to 747) mL versus 400 (269 to 565) mL; p = 0.4; Table 5). Of the patients requiring PRC transfusion, 39% had medical treatment (61% had surgical treatment), but there was also no difference in the PRC transfusion requirements during DrotAA between medical and surgical patients (860 (548 to 928) mL versus 747 (450 to 1117) mL; p = 0.86). Similarly there was no difference in the minimum value of haemoglobin during DrotAA and in the post-DrotAA period (Table 4).

**Table 4:** Clotting and haematological parameters during and post-DrotAA infusion.

**Variable**	**DrotAA median (IQR)**	**Post-DrotAA Median (IQR)**	**p-value**
Hb_Max _(g/dL)	10.1 (9.57 to 11.0)	9.45 (8.9 to 10.3)	< 0.01*
Hb_Min_, (g/dL)	8.7 (8.1 to 9.3)	8.3 (7.4 to 9.1)	0.1
PLT_Max_,	104 (64 to 177)	110 (85 to 166)	0.8
PLT_Min_	88 (41 to 164)	99 (60 to 160)	0.8
Fibrinogen_Max_	5.1 (3.8 to 6.7)	4.4 (3.1 to 6.0)	0.07
Fibrinogen_Min_	4.85 (3.4 to 6.1)	4.4 (3.2 to 6.0)	0.3
INR_Max_	1.43 (1.3 to 1.7)	1.3 (1.2 to 1.8)	0.047*
INR_Min_	1.3 (1.2 to 1.5)	1.3 (1.1 to 1.6)	0.8
APTT_Max_, s	57 (48.6 to 69.2)	43.8 (37 to 62)	< 0.01*
APTT_Min_, s	51 (43.2 to 59)	44 (37 to 62)	0.05

Data are expressed as maximum and minimum median values (interquartile range).

APTT = activated partial thromboplastin time; DrotAA = drotrecogin alfa (activated), Hb = haemoglobin, INR = international normalized ratio; PLT = platelets.

**Table 5:** Blood product transfusion during and post-DrotAA infusion.

**Variable**	**DrotAA median (IQR)**	**Post-DrotAA median (IQR)**	**p-value**
PRC transfused, mL	549 (292 to 747)	400 (269 to 565)	0.4
Platelets transfused, mL	507 (313 to 597)	290 (285 to 295)	0.15
FFP transfused, mL	1566 (574 to 1884)	819 (807 to 955)	0.35

Data are expressed as median values (interquartile range).

DrotAA = drotrecogin alfa (activated), FFP = fresh frozen plasmam PRC= packed red cell.

A larger proportion of patients required platelet transfusion during DrotAA infusion compared with post-DrotAA on heparin (38.4% versus 14.2%; p = 0.2, Fisher's test), with a median (IQR) volume of platelets transfused during DrotAA of 507 (313 to 597) mL compared with the post-DrotAA period of 290 (285 to 295) mL (p = 0.15), (Table 5). Similarly, there was a difference in the number of patients receiving fresh frozen plasma during DrotAA (17% versus 8%), with a median (IQR) of fresh frozen plasma transfused of 1566 (574 to 1884) mL versus 819 (807 to 955) mL in the post-DrotAA period (Table 5). Transfusion of fresh frozen plasma was associated with higher INR levels of 1.43 (1.3 to 1.7) during DrotAA versus 1.30 (1.2 to 1.8) during the post-DrotAA period (p = 0.047) and a more prolonged APTT of 56.9 (48.6 to 69.2) seconds during DrotAA versus 43.8 (37 to 69.9) seconds during the post-DrotAA period (p < 0.01; Table 4).

## Discussion

In 2002, when DrotAA was licensed, no strict protocols or guidelines were available in the literature on how to manage RRT in patients receiving DrotAA. In our institution, clinicians made decisions about anticoagulation in these patients according to experience and after taking into account the platelet count, clotting times and filter parameters. This is reflected in the variety of anticoagulation regimens used in our dataset.

Our data suggest that additional anticoagulation during DrotAA infusion does not improve filter survival time. Also, the lifespan of the circuit during DrotAA is similar to that seen in the presence or absence of additional anticoagulation while on DrotAA and with standard anticoagulation in the 96 hours immediately after DrotAA therapy. Therefore, there is probably no need for routine additional anticoagulation to preserve circuit function and this became our practice after 2006.

The data also suggest that the use of DrotAA in patients with severe sepsis requiring RRT appears safe and is not associated with an increased need for PRC transfusion or important bleeding events.

During the DrotAA infusion, there was a greater, albeit not statistically significant, volume of platelets and fresh frozen plasma transfused compared with the post-DrotAA period. It is difficult to understand whether this difference can be entirely attributed to DrotAA, or whether it is multi-factorial. Other possible explanations include variable thresholds among clinicians to correct the commonly observed increased clotting times induced by DrotAA, or attempts to correct a bleeding event or a sepsis-induced coagulopathy.

There is minimal data on how to manage anticoagulation in patients on RRT during the infusion DrotAA therapy and in clinical practice the choice of RRT modalities and anticoagulation is dependent on expert opinion, and the clinician's level of experience with both treatments. Our data shows that DrotAA is as effective as heparin in terms of FST, and these data are in accordance with a previous report on three patients receiving DrotAA while on RRT [11]. Several small studies have also confirmed that in patients at high risk of bleeding and with some degree of coagulopathy, RRT in the absence of anticoagulation does not result in a shorter circuit survival time [12,13]. Furthermore, the presence of sepsis-induced coagulopathy and the effects of DrotAA on the APTT make the monitoring of anticoagulation unreliable and can increase the risk of inappropriate anticoagulation. Knowledge of the effect of DrotAA on FST is also important in the presence of thrombocytopenia, which may caution the concomitant use of heparin. Anticoagulation can be recommenced, as per standard practice, when the infusion of DrotAA has been completed [9].

In this study we found that our FSTs were generally short (less than 24 hours). This duration was not solely attributable to filter clotting, but it was often (60.7% of circuit changes during DrotAA and 63.6% post-DrotAA) due to investigations (e.g., computed tomography scan) or a surgical procedure.

Although our study shows that no additional anticoagulation is necessary for filter survival, it is now probably advisable to continue treatment with prophylactic heparin in all patients who were already receiving it before RRT, as suggested by the Xigris and PRophylactic hEparin in Severe Sepsis (XPRESS) study [14,15]. The decision to use heparin should be taken independently from the decision to use DrotAA and should take into consideration the bleeding risk of each individual patient.

Our study has the limitation of being a retrospective analysis in a small number of patients; however, this is an area of practice that is clinically important and in which it is difficult for individual clinicians working in smaller centres to gain sufficient experience. Another potential confounder is that during the two periods of time the patients are clearly at different stages of evolution of the sepsis episode. One therefore might expect different patterns of coagulation activity between the two groups. Even so, given these important caveats, it is valuable to be able to show that anticoagulation for RRT can be carried out safely with DrotAA alone and that additional anticoagulation is not routinely necessary.

## Conclusion

Additional anticoagulation in patients receiving RRT during DrotAA infusion is not routinely necessary and does not improve FST. The use of DrotAA in patients with severe sepsis requiring RRT is safe and is not associated with an increased need for PRC transfusion. Interpretation of the effects of DrotAA on platelet and fresh frozen plasma transfusion are confounded by the presence of sepsis-induced coagulopathy.

## Key messages

• Additional anticoagulation in patients receiving RRT during DrotAA infusion is not routinely necessary and does not improve FST.

• The use of DrotAA in patients with severe sepsis requiring RRT is safe and is not associated with an increased need for PRC transfusion.

• Interpretation of the effects of DrotAA on platelet and fresh frozen plasma transfusion requirements are confounded by the presence sepsis-induced coagulopathy.

## Abbreviations

APTT: activated partial thromboplastin time; DrotAA: Drotrecogin alfa (activated); FST: filter survival time; ICU: intensive care unit; INR: international normalized ratio; IQR: interquartile range; PRC: packed red cells; RRT: renal replacement therapy.

## Competing interests

DW has received lecture fees from Eli Lilly and Company (the manufacturer of drotrecogin alfa) and has acted as a consultant to Eli Lilly and Company over the past six years. RB has acted as a consultant for Eli Lilly and Company, and Guy's and St Thomas' NHS Foundation Trust has billed speaking fees and honoraria on his behalf. The other authors declare that they have no competing interests.

## Authors' contributions

LC made substantial contributions to the design, acquisition, analysis and interpretation of data, performed the statistical analysis and drafted the manuscript. EC and EM made substantial contributions to the design, acquisition and analysis of the data and critically revised the manuscript. JS and KL made substantial contributions to the acquisition of data. RB and DW made substantial contributions by critically revising the manuscript, were involved in the design, analysis, drafting of the manuscript and gave final approval of the version to be published. All authors read and approved the final manuscript.

## Supplementary Material

Additional file 1 Word file containing a listing of indications and contraindications to the use of Drotrecogin alfa (activated). Click here for file
