# Supplementary material for: Filter survival time and requirement of blood products in patients with severe sepsis receiving drotrecogin alfa (activated) and requiring renal replacement therapy
Source: Crit Care. 2008 Dec 18;12(6):R163. doi: 10.1186/cc7163 (PMC2646328; doi:10.1186/cc7163)
Supplement: Additional file 1 — Word file containing a listing of indications and contraindications to the use of Drotrecogin alfa (activated). [file cc7163-S1.doc]

**Additional data**

**Indications and contraindications to the use of Drotrecogian alfa (activated)**

**Inclusion Criteria**

1. Suspected or proven infection (e.g. pneumonia, perforated viscus) **AND**
2. Presence of ≥ 2 **sustained** organ dysfunctions induced by sepsis:
   1. Hypotension *despite adequate fluid* and/or requirement for vasopressors to achieve satisfactory blood pressure
   2. Persistent oliguria *despite adequate fluid*
   3. Acute hypoxaemia: PaO_2_/FiO_2_ ratio ≤ 33 kPa (≤ 26.6 in the setting of pneumonia)
   4. Platelet count ≤ 80 **or** a 50% decrease over last 3 days
   5. Sepsis induced metabolic acidosis or elevated lactate
3. **Exclusion Criteria**
   - 1. Active internal bleeding
     2. GI bleed in last 6 weeks where definitive treatment has not been performed
     3. Patients with intracranial pathology e.g. neoplasm
     4. Intracranial/spinal surgery within previous 3 months
     5. History of recent severe head trauma or haemorrhagic stroke
     6. Trauma at increased risk of bleeding
     7. Surgery within the last 12 hours
     8. Concurrent heparin therapy ≥ 15 IU/kg/hr
     9. Known bleeding diathesis (except for coagulopathy related to sepsis)
     10. Platelet count <30, even if count is increased after transfusions§
     11. Presence of an epidural catheter
     12. Patients who are not expected survive 24 hours
     13. Prior cardiac arrest without demonstrable neurological recovery
     14. Patients with uncorrectable medical condition©: e.g.
4. A) Severe COPD; NYHA IV cardiac failure; End-stage neurological disease; Chronic severe hepatic disease; AIDS
   1. Advanced cancer (e.g. Duke’s C carcinoma of colon) or those currently receiving chemotherapy

**Notes**

1. Note that in the SPC, warfarin, aspirin, clopidogrel and thrombolytics are not contraindications to the use Xigris_®_, but are special warnings.
2. § Severe thrombocytopaenia is included in the contraindication section of the SPC. (In the FDA label it is *not* a contraindication).
3. © In the PROWESS/ENHANCE studies the strict APACHE II definitions were used to exclude patients with chronic health. Despite the lack of evidence-base in this group, there may be those patients (e.g. stable chronic renal failure, successful renal transplant) that after careful individual consideration may benefit from Xigris^®^.
